# O.4.3-2 Teaching movement and physical activity in Early Childhood Education and Care, an obvious and important objective, or a challenging and provocative task for educators?

**DOI:** 10.1093/eurpub/ckad133.188

**Published:** 2023-09-11

**Authors:** Ann-Christin Sollerhed

**Affiliations:** Kristianstad University, Sweden; ann-christin.sollerhed@hkr.se

## Abstract

Movement and physical activity (MoPA) are critical to children’s health and development. Most children aged 1-5 years are enrolled in Early Childhood Education and Care (ECEC) in Sweden. High expectations are placed on educators to deliver education of sufficient quality to support children’s development.

**Purpose:**

The aim of the 18-month-long action-based study was to investigate how 88 ECEC educators in five preschools experienced their MoPA-teaching.

**Methods:**

The educators planned and implemented MoPA sessions in a trial-and-error manner among children. They filmed sequences from the sessions, which were shown in focus groups led by the researcher. The film sequences were the starting point for the collegial discussions.

**Results:**

Content analysis of the transcribed focus group discussions revealed three themes with each two subcategories (in brackets): 1. Teaching aspects (educator’s competence; educator’s role modeling);2. Educational aspects (children’s development; children’s health and wellbeing); 3. Structural aspects (curriculum; environment).

Most educators perceived their competence to teach MoPA as insufficient, which was perceived as a troublesome barrier. The teaching sessions were often replaced with free play. During the project the educators became aware of children’s different MoPA-levels and that free play did not always increase the children’s motor skills. Despite this awareness, most of the educators were not prepared to leave their comfort zones, to invest time, work, or effort to improve the MoPA-education. Most of the educators felt it was difficult to assume the role of a physically active role model for the children. They demonstrated the need for continuous education to enhance the pedagogic content knowledge for adequate MoPA-teaching. Many educators demonstrated that teaching MoPA was seen as an arduous task, although they were aware of the importance of children’s motor skill learning and physical activity for a healthy development. They thought it was difficult to keep moving children in order and they preferred sedentary activities where their leadership was perceived as sufficient.

**Conclusions:**

The pedagogical content knowledge in MoPA is low among ECEC-educators and the MoPA-teaching is often seen as difficult to implement and is therefore unfavorably treated in ECEC, which may have negative implications for children’s actual and future health.

